# Thoracic aortic aneurysm combined with intracranial vascular abnormalities caused by dual mutations in *MYLK* and *FBN2*: a case report

**DOI:** 10.3389/fgene.2025.1672342

**Published:** 2025-10-13

**Authors:** Maorong Cai, Yang Liu, Zhaodi Liao, Yiping Wu, Jiantong Jiao

**Affiliations:** ^1^ The Affiliated Wuxi People’s Hospital of Nanjing Medical University, Wuxi People’s Hospital, Wuxi Medical Center, Nanjing Medical University, Nanjing, China; ^2^ Department of Neurosurgery, The Affiliated Wuxi People’s Hospital of Nanjing Medical University, Wuxi People’s Hospital, Wuxi Medical Center, Nanjing Medical University, Wuxi, Jiangsu, China

**Keywords:** thoracic aortic aneurysm/dissection (TAA/D), intracranial vascular abnormalities, whole-exome sequencing (WES), MYLK mutation, FBN2 mutation, dual gene mutations, intracranial vascular screening

## Abstract

**Objective:**

To perform genetic testing on a patient with ruptured vertebral artery aneurysm and subarachnoid hemorrhage who was also found to have a thoracic aortic aneurysm/dissection (TAA/D) during preoperative evaluation, along with their family members. The aim was to identify potential pathogenic gene variants, analyze the inheritance pattern, and investigate the association with coexisting intracranial and aortic vascular abnormalities.

**Methods:**

Intracranial vascular lesions (ruptured vertebral artery aneurysm and subarachnoid hemorrhage) were confirmed via computed tomography (CT), computed tomography angiography (CTA), and digital subtraction angiography (DSA). Whole-exome sequencing (WES) via next-generation sequencing (NGS) was conducted on the proband and family members to identify pathogenic gene mutations associated with thoracic aortic aneurysm/dissection (TAA/D) and intracranial vascular abnormalities, thereby elucidating the underlying genetic mechanisms.

**Results:**

This study reports the management of a patient with a ruptured vertebral artery aneurysm, subarachnoid hemorrhage, and concomitant TAA/D incidentally detected during preoperative evaluation. Imaging studies demonstrated occlusion at the vertebral-basilar junction, with the basilar artery being perfused by the anterior circulation. An aneurysm was identified at the vertebral artery confluence, and the right vertebral artery was found to supply the left vertebral artery, left subclavian artery, and descending aorta. The surgical procedure was performed successfully under general anesthesia, and the patient was transferred to the ward in stable condition. NGS revealed two heterozygous mutations in the patient: a maternally inherited *MYLK* variant (NM_053025.4): c.834_835insGTA (p.Val278dup) and a paternally inherited *FBN2* variant (NM_001999.4): c.1478A>G (p.Gln493Arg). Sequence analysis identified novel mutation sites within both genes, which may contribute to the patient’s combined vascular phenotype. Following the procedure, the patient maintained hemodynamic stability and recovered well after discharge without notable cardiopulmonary abnormalities or surgery-related complications.

**Conclusion:**

Our findings expand the mutational spectrum of non-syndromic familial thoracic aortic aneurysm/dissection (TAA/D), highlighting that associated gene mutations may also predispose to intracranial vascular abnormalities. We therefore recommend routine intracranial vascular screening (e.g., CTA/DSA) for patients with familial TAA/D to detect potential intracranial lesions. This case underscores the critical need for comprehensive clinical-genetic evaluation to facilitate early diagnosis and timely intervention, which may improve patient outcomes and reduce morbidity.

## 1 Introduction

Thoracic Aortic Aneurysm/Dissection (TAAD) is a life-threatening vascular disorders characterized by aortic wall dilatation and potential rupture, with a prevalence of 0.16%–0.34% and an annual incidence of 3.5–10 cases per 100,000 individuals (Berger et al., 2025). Clinically, TAAD manifests as progressive aortic bulging, which may evolve into dissection—a condition marked by insidious onset, acute decompensation, and high mortality, necessitating urgent interventio (Ziganshin and Elefteriades, 2014). Genetically, TAAD is classified into familial (FTAAD, ∼20% of cases) and sporadic forms, with FTAAD typically following an autosomal dominant inheritance pattern. FTAAD patients present at a younger age (58.2 vs. 65.7 years) and exhibit faster aortic expansion (0.21 vs. 0.16 cm/year) than sporadic cases (Ostberg et al., 2020; Hannuksela et al., 2015).

The ClinGen Aortopathy Working Group has identified over 20 TAAD-associated risk genes, with pathogenic mechanisms primarily involving: 1) impaired vascular smooth muscle cell contractility (e.g., *ACTA2*, *MYH11*, *MYLK*, *PRKG1* mutations) and 2) dysregulated TGF-β signaling (e.g., *FBN1*, *TGFBR1*, *TGFBR2* mutations) (Duarte et al., 2023). Notably, *ACTA2*, *MYH11*, *MYLK*, and *PRKG1* mutations are particularly relevant, as they can induce dissection at aortic diameters <5.0 cm (Rombouts et al., 2022). Genetic heterogeneity further influences phenotypic severity: the *MYLK* mutation spectrum, for instance, shows that missense variants confer earlier disease onset and higher risk of aortic symptoms (38% penetrance) compared to null mutations (Wallace et al., 2019).

A growing body of evidence highlights the multisystem vascular involvement in FTAAD, with patients frequently presenting with concomitant abdominal aortic aneurysms or cerebral aneurysms (Isselbacher et al., 2016; Hamilton, 2011). This observation supports a shared genetic network governing vascular development, though clinical heterogeneity remains a challenge—most FTAAD cases are asymptomatic until acute events (e.g., dissection, rupture) or intracranial hemorrhage, and some are incidentally detected via imaging (Abdelrahman et al., 2023). Such heterogeneity implies that polygenic interactions, beyond established monogenic pathways, may drive disease progression (Liu et al., 2012) This is particularly evident in cases with combined aortic and cerebrovascular malformations (e.g., vertebral artery remodeling), where monogenic models fail to explain the pathology. While dominant mutations in *ACTA2* and *MYH11* have clarified, the role of biallelic or compound heterozygous mutations remains underexplored—representing a critical gap in understanding TAAD with cerebrovascular comorbidities (Keravnou et al., 2018; Burger et al., 2021).

Here, we report a rare non-syndromic FTAAD case with vertebral artery anatomical malformation. Whole-exome sequencing and familial segregation analysis revealed the patient harbored compound heterozygous mutations: *MYLK* (NM_053025.4): c.834_835insGTA (p.Val278dup) (maternally inherited), and *FBN2* (NM_001999.4): c.1478A>G (p.Gln493Arg) (paternally inherited). The co-occurrence of thoracic aortic aneurysm and aberrant vertebral artery perfusion (right vertebral artery supplying the left subclavian artery and descending aorta) suggests a mechanistic model where *MYLK*-mediated contractility defects and *FBN2*-dependent elasticity impairment act additively to disrupt vascular mechanohomeostasis (Humphrey and Schwartz, 2021). This discovery not only expands the mutational spectrum of non-syndromic TAAD but also provides a novel molecular framework for combined intracranial-aortic vascular pathologies, underscoring the need for systematic cerebrovascular screening in FTAAD families.

## 2 Materials and methods

### 2.1 Ethics Statement

This study was reviewed and approved by the Ethics Committee of Wuxi People’s Hospital of Nanjing Medical University. Written informed consent was obtained from all participants.

### 2.2 DNA extraction and analysis

Genomic DNA was extracted from peripheral blood samples using the DNeasy Blood Kit (Qiagen) according to the manufacturer’s instructions. For each sample, 1–2 mg genomic DNA was fragmented by M220 Focused-ultrasonicator (Covaris) into ∼250 bp. A whole-genome library was prepared using the TargetCap Core Exome Panel V3.0 (Boke Bioscience, Inc.) according to the manufacturer’s protocol. Captured libraries were amplified in KAPA HiFi HotStart ReadyMix (KAPA Biosystems) and purified using Agencourt AMPure XP beads. Enriched libraries were sequenced in a 150-bp paired-end mode on a DNBSEQ-T7 platform (MGI Tech. Co. Ltd., Shenzhen, China) with an average coverage depth of >100×. Finally, the original image data were converted to raw data in FASTQ format after base calling.

### 2.3 Variant calling and annotation

Raw sequencing reads were first merged and subjected to quality control using fastp (v0.20.1) to remove low-quality reads and adapter sequences. Clean reads were then aligned to the human reference genome (GRCh37/hg19) using the Sentieon implementation of BWA with default parameters. The resulting BAM files were sorted, deduplicated, realigned around indels, and subjected to base quality score recalibration using Sentieon tools following GATK best practices.

Single nucleotide variants (SNVs) and small insertions/deletions (indels) were identified using GATK HaplotypeCaller. Variant calls were further filtered and annotated using an in-house pipeline, which integrates multiple annotation tools and databases, including ANNOVAR (for functional annotation and population frequency, using 1000 Genomes, ExAC, gnomAD, dbSNP, ClinVar, dbscSNV, HGMD, LOVD, OMIM, Orphanet, GSDB, COSMIC, etc.), VEP (Variant Effect Predictor, for transcript and protein effect prediction), and InterVar (for ACMG guideline-based variant classification). Copy number variants (CNVs) were detected using both GATK GermlineCNVCaller (v4.5) and XHMM, and the results were integrated. All identified variants were classified according to the American College of Medical Genetics and Genomics (ACMG) guidelines and the Clinical Genome Resource (ClinGen) recommendations, into five categories: pathogenic, likely pathogenic, variant of uncertain significance (VUS), likely benign, or benign. The final variant interpretation was performed by a multidisciplinary team, integrating genetic, clinical, and family information.

## 3 Results

### 3.1 Clinical history

**FIGURE 1:**
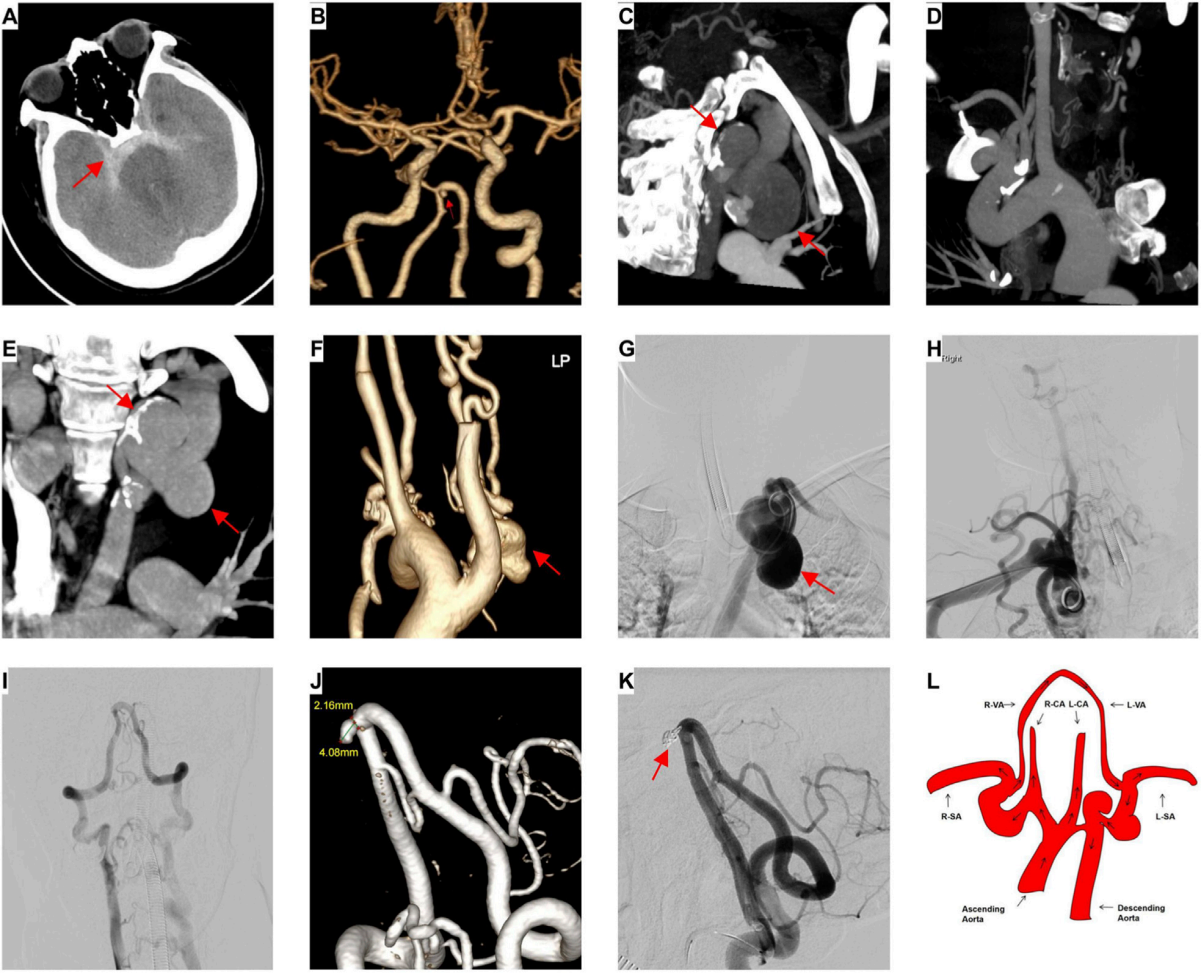
Imaging manifestations of the patient. **(A)** Head CT demonstrates subarachnoid hemorrhage with an anterior brainstem hematoma (red arrow). **(B)** Head CTA shows a columnar protrusion at the vertebral artery confluence (red arrow) with discontinuity between the vertebral and basilar arteries. **(C–F)** Chest CTA reveals a thoracic aortic aneurysm in the patient (red arrow). **(G)** DSA via a femoral artery approach shows localized saccular dilation of the thoracic aorta (red arrow). **(H)** DSA image of the right subclavian artery obtained via a right transradial approach. **(I)** Right vertebral artery DSA shows convergence of the bilateral vertebral arteries intracranially, with no visualization of the basilar artery. **(J)** Three-dimensional reconstruction and morphometric analysis of the aneurysm. **(K)** DSA confirms dense packing of the aneurysm, with satisfactory stent position and wall apposition (red arrow). **(L)** Schematic diagram of the patient’s abnormal blood flow distribution.R-SA:Right Subclavian Artery; L-SA:Left Subclavian Artery; R-CCA:Right Common Carotid Artery; L-CCA:Left Common Carotid Artery; R-VA:Right Vertebral Artery; L-VA:Left Vertebral Artery.

The patient was a 33-year-old woman who presented to the emergency department with a 4-h history of sudden-onset severe headache, nausea, and vomiting.She has no history of smoking and denies any history of hypertension or diabetes. There is no history of dyslipidemia (total cholesterol, LDL-C, HDL-C, and triglycerides are all within normal range), nor is there a family history of premature cardiovascular disease. On admission, vital signs showed left upper arm blood pressure (BP) 93/73 mmHg, right upper arm BP 145/98 mmHg, heart rate 115 bpm, and respiratory rate 37 breaths/min. Laboratory results included hemoglobin 9 g/dL, white blood cell count 16 × 10^9^/L, erythrocyte sedimentation rate 12 mm/h, C-reactive protein 2 mg/dL, D-dimer 942 ng/mL, B-type natriuretic peptide (BNP) 1,000 pg/mL, and troponin T 0.05 ng/mL. Head CT indicated subarachnoid hemorrhage (SAH) with brainstem anterior hematoma (Figure 1A), and subsequent head CTA revealed a columnar protrusion at the vertebral artery confluence, and discontinuity between the vertebral artery and the basilar artery (Figure 1B). The patient had a normal phenotype with no stigmata of connective tissue disorders, no significant medical history, and a prior pregnancy notable for inter-arm BP discrepancy prompting thoracic-abdominal CTA, which incidentally detected a thoracic aortic aneurysm (Figures 1C–F). Family history was negative for aortic disease, and growth and development were unremarkable.

Given the diagnosis, the patient underwent emergency cerebral angiography. This confirmed the presence of a saccular aneurysm at the vertebral artery confluence. Subsequently, a single-stage stent-assisted coiling procedure was performed. During the procedure, an interventional access was established via the right femoral artery. Attempts to advance the angiographic catheter to the aortic arch encountered resistance. Contrast injection revealed a localized spherical dilatation of the thoracic aorta, with blood supply originating from the left subclavian artery; no patent channel toward the ascending aorta was identified, suggesting a thoracic aortic aneurysm (Figure 1G). DSA was performed via a right transradial approach. Angiography of the right subclavian artery revealed multiple local vascular variations (Figure 1H). The catheter was selectively advanced into the right vertebral artery. Subsequent angiography showed that the intracranial segments of the bilateral vertebral arteries converged; however, continuity with the basilar artery was lost, and the right vertebral artery supplied blood flow to the left vertebral artery (Figure 1I). Three-dimensional rotational angiography confirmed an aneurysm at the terminal point of the right vertebral artery. The aneurysm was irregular in shape, with a neck measuring 2.16 mm and a dome height of 4.08 mm (Figure 1J). These findings were consistent with the preoperative CTA results. The angiographic findings were discussed with the patient’s family. Subsequently, the preoperatively planned aneurysm embolization procedure was performed. This aneurysm was successfully treated with stent-assisted coiling. Post-procedural angiography confirmed dense packing of the aneurysm and satisfactory positioning and wall apposition of the stent (Figure 1K). The procedure was uneventful, general anesthesia was effective, and the patient recovered well postoperatively. Integrating the findings from the previous thoracic CTA and the significant inter-arm blood pressure difference, the diagnosis was a thoracic aortic aneurysm involving the proximal segment. This was associated with aberrant blood flow distribution: the ascending aorta had only a small channel connecting to the thoracic aorta, and blood flow from the right vertebral artery passed retrograde via the left vertebral artery to supply the left subclavian artery and the descending aorta (Figure 1L).

### 3.2 Targeted sequencing and genetic analysis

**FIGURE 2:**
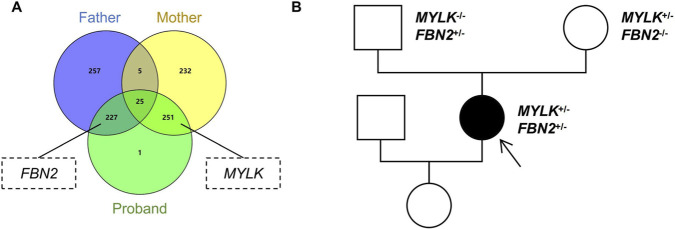
NGS testing of the case family. **(A)** A Venn diagram of mutated genes in the proband and her parents, with genes potentially associated with vascular diseases annotated. **(B)** Family pedigree. White circles/squares indicate unaffected family members, and the black arrow indicates the proband.

Considering the patient’s young age and idiopathic presentation, we suspected the presence of pathogenic genetic variants underlying her condition. Therefore, we collected blood samples from the patient and their parents and conducted whole-exome sequencing (NGS). The sequencing results identified germline mutations in 504 (patient), 514 (father), and 513 (mother) genes. Comparative analysis showed 252 shared genes between patient and father, 276 between patient and mother, and 25 common to all three, indicating parental inheritance. Screening of genes known to be potentially associated with vascular diseases revealed that the patient carried heterozygous mutations in *FBN2* (NM_001999.4): c.1478A>G (p.Gln493Arg) (paternally inherited) and *MYLK* (NM_053025.4): c.834_835insGTA (p.Val278dup) (maternally inherited) (Figure 2A). However, imaging screening confirmed that both parents were unaffected and without a clinical history of TAAD (Figure 2B).

**FIGURE 3:**
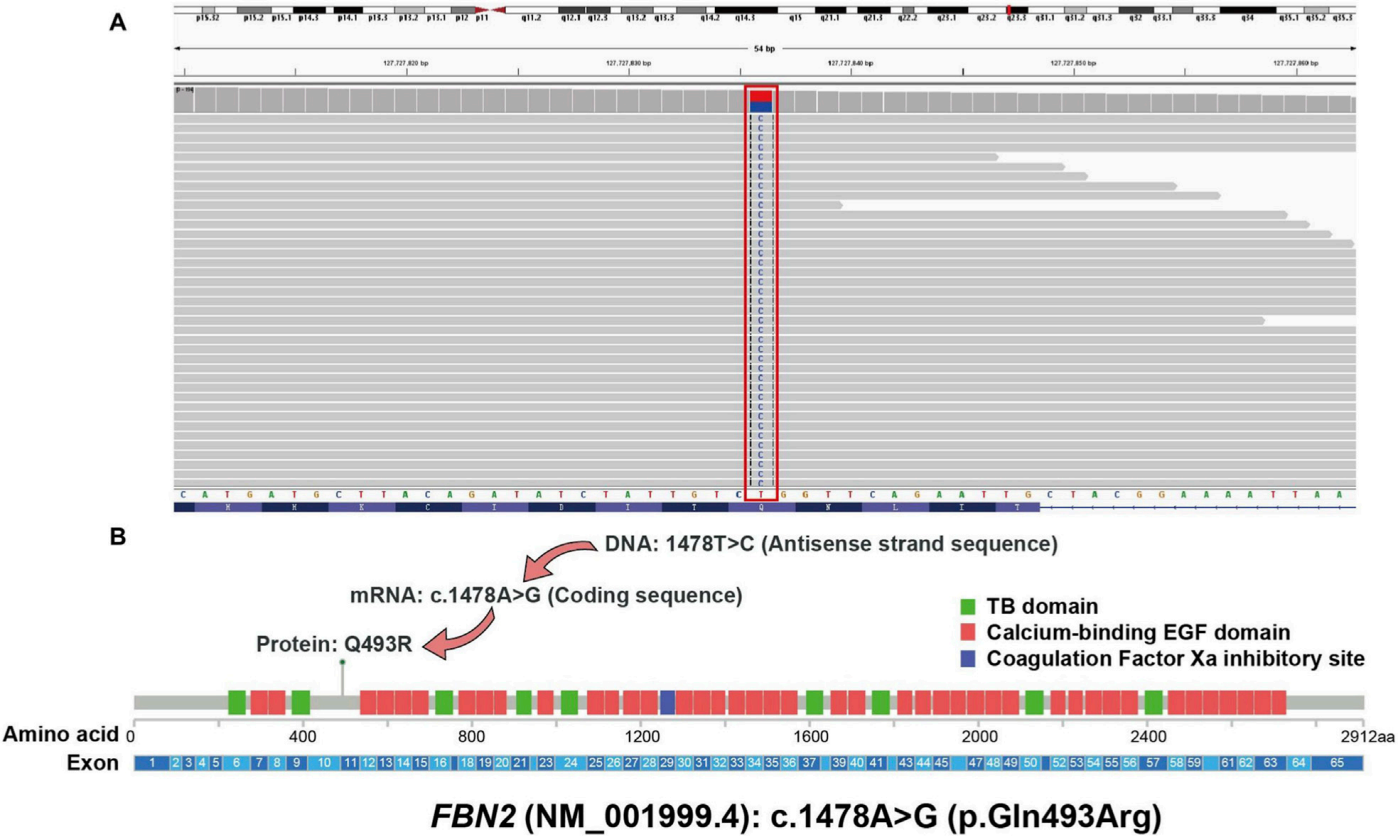
Visualization of *FBN2* Gene Mutation. Figure 3 includes two parts for the variant of *FBN2* (NM_001999.4): c.1478A>G (p.Gln493Arg): **(A)** A screenshot from Integrative Genomics Viewer (IGV) showing next-generation sequencing (NGS) reads, confirming the heterozygous germline missense variant in the *FBN2* gene; **(B)** A schematic diagram illustrating how the DNA-level variation (c.1478A>G) results in the corresponding amino acid change (p.Gln493Arg) in the *FBN2* protein, as well as the location of this protein-level variant relative to different functional domains of the full-length *FBN2* protein.

A novel heterozygous germline missense variant in *FBN2* (NM_001999.4): c.1478A>G (p.Gln493Arg) was detected in the proband’s sample (Figures 3A,B).

**FIGURE 4:**
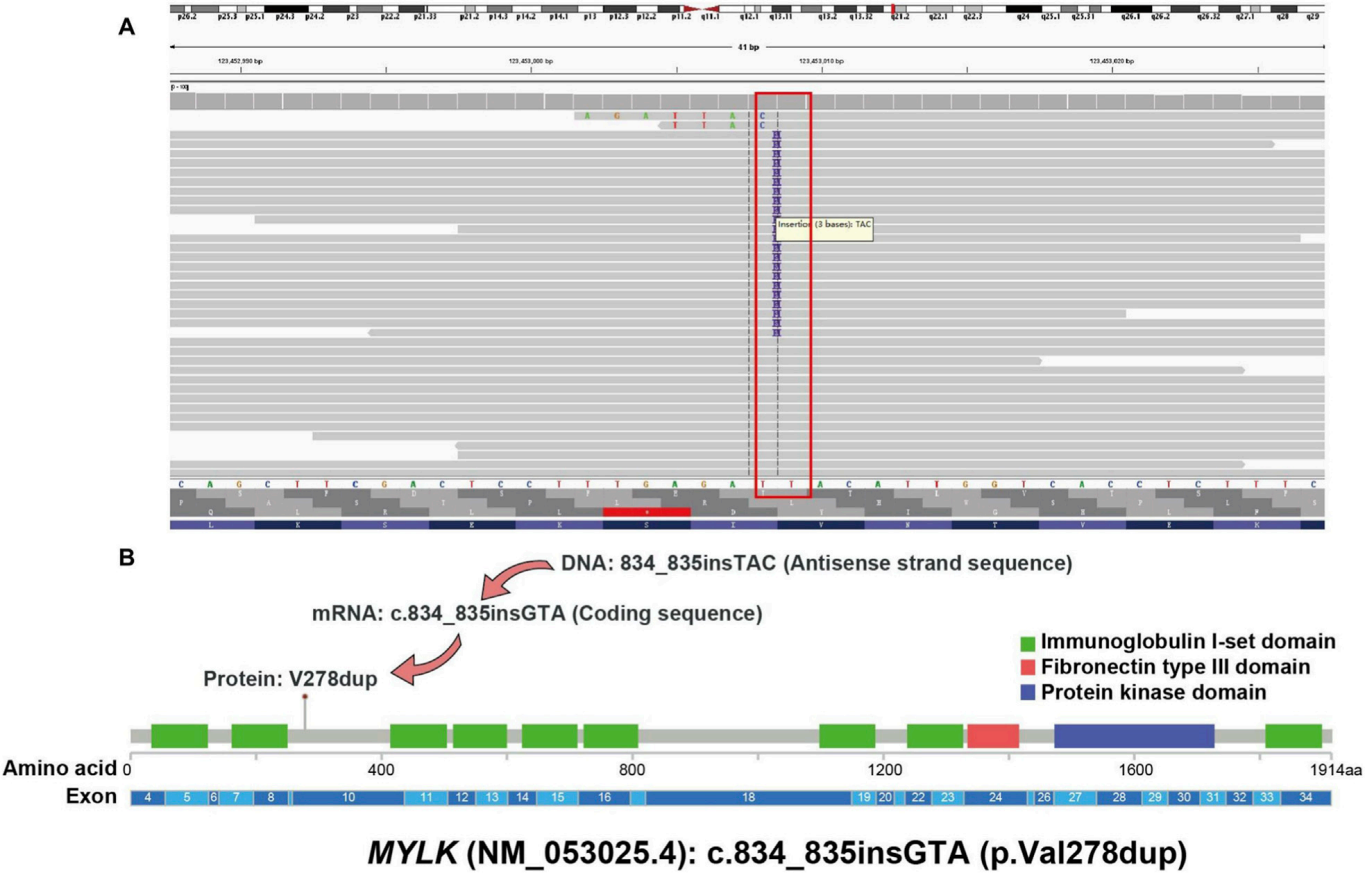
Visualization of *MYLK* Gene Mutation. Figure 4 includes two parts for the variant of *MYLK* (NM_053025.4): c.834_835insGTA (p.Val278dup): **(A)** A screenshot from Integrative Genomics Viewer (IGV) showing next-generation sequencing (NGS) reads, confirming the heterozygous germline non-frameshift duplication variant in the *MYLK* gene; **(B)** A schematic diagram illustrating how the DNA-level variation (c.834_835insGTA) results in the corresponding amino acid change (p.Val278dup) in the *MYLK* protein, as well as the location of this protein-level variant relative to different functional domains of the full-length *MYLK* protein.

This mutation is absent in population databases (1000G, ExAC, gnomAD), satisfying the PM2_Supporting criterion of ACMG guidelines. Functional prediction tools (LRT, MutationTaster, Gerp+, CADD) all consistently support its potential pathogenicity. Additionally, a novel heterozygous germline non-frameshift duplication variant in *MYLK* (NM_053025.4): c.834_835insGTA (p.Val278dup) was identified; this variant is located in a non-repetitive genomic region, causes protein length alteration (satisfying the PM4 criterion of ACMG guidelines), and is also absent from the above-mentioned databases (meeting the PM2_Supporting criterion)(Figures 4A,B). Taken together, these findings suggest that *FBN2* (NM_001999.4): c.1478A>G (p.Gln493Arg) and *MYLK* (NM_053025.4): c.834_835insGTA (p.Val278dup) may serve as key genetic determinants contributing to the patient’s early-onset vertebral artery aneurysm and heightened risk of TAA/D (Table 1).

**TABLE 1:** NGS and family validation confirmed that the proband had mutations in the *MYLK* and *FBN2* genes, inherited from the father and mother, respectively.

Gene	Chromosomal location	Genetic subregion	Nucleotide change	Associated diseases	Mode of inheritance	Zygosity	Inheritance
*FBN2* (NM_001999.4)	chr5:127,727,836	exon11	c.1478A>G (p.Q493R)	Congenital Contractural Arachnodactyly (OMIM:121050)	AD	Heterozygous	Father
*MYLK* (NM_053025.4)	chr3:123453008	exon10	c.832_834dup (p.V278dup)	Familial Thoracic Aortic Aneurysm and Dissection Type 7 (OMIM:613780)	AD	Heterozygous	Mother

## 4 Discussion

In this report, we presented the endovascular management of a patient with a ruptured vertebral artery aneurysm accompanied by subarachnoid hemorrhage (SAH) and concurrent thoracic aortic aneurysm/dissection (TAAD). Whole-exome sequencing identified a novel heterozygous germline missense variant in *FBN2* (NM_001999.4): c.1478A>G (p.Gln493Arg) and a novel heterozygous germline non-frameshift duplication in *MYLK* (NM_053025.4): c.834_835insGTA (p.Val278dup). Although neither variant has been previously reported in the literature, functional predictions suggest they may act synergistically to disrupt vascular wall integrity, ultimately leading to aneurysm formation at multiple sites. This finding underscores the critical importance of systematic genetic testing: clinically significant hereditary variants may be identified even in patients lacking a typical family history (Yamaguchi et al., 2025).

The *MYLK* gene is the causative gene for Familial Thoracic Aortic Aneurysm and Dissection Type 7 (FTAAD7, OMIM:613780), following an autosomal dominant inheritance pattern with incomplete penetrance (Shalata et al., 2018). *MYLK* encodes smooth muscle myosin light chain kinase (*MLCK*), a key regulatory protein for smooth muscle cell contraction. *MLCK* binds to the calmodulin complex to phosphorylate the 20 kDa regulatory light chain of myosin, thereby increasing myosin II ATPase activity. This releases energy to drive the cross-bridge cycling of β-myosin heavy chain with α-actin, resulting in smooth muscle contraction (Khapchaev and Shirinsky, 2016). *MYLK* mutations can lead to dysfunction of smooth muscle contraction through a loss-of-function mechanism. For instance, *MYLK* nonsense mutations have been reported to disrupt the kinase domain of *MLCK* via premature termination of translation, resulting in insufficient phosphorylation of myosin light chains and failure of smooth muscle cell contraction, thereby predisposing to thoracic aortic aneurysms and dissections (Luyckx et al., 2017). Consistent with this, *MYLK* knock-in mice carrying kinase-inactivating mutations develop ascending aortic aneurysms before 6 months of age, further confirming that defective smooth muscle cell contraction is sufficient to trigger aneurysmal dilation (Wang et al., 2010). This functional impairment is particularly critical in the aorta, where rhythmic smooth muscle contraction works in concert with elastic fibers to maintain vascular wall tension and elasticity; when this regulation is disrupted, the vessel wall becomes unable to withstand hemodynamic stress, ultimately leading to aneurysmal dilation or dissection (Milewicz et al., 2017). Notably, the impact of *MYLK* mutations is not limited to large vessels, as loss of function can also impair intestinal smooth muscle contraction, causing megacystis microcolon intestinal hypoperistalsis syndrome, which further underscores the pivotal role of this gene in smooth muscle function (Halim et al., 2017).

The *FBN2* gene is the causative gene for Congenital Contractural Arachnodactyly (CCA, OMIM:121050), inherited in an autosomal dominant manner with near-complete penetrance (Xu et al., 2020). *FBN2* encodes Fibrillin-2, a major component of connective tissue microfibrils. It plays a crucial role in the early stages of elastic fiber assembly, providing structural support, strength, and flexibility to connective tissues (Mahdizadehi et al., 2023). When an *FBN2* mutation occurs, the resulting abnormal fibrillin-2 protein incorporates into microfibrils through a dominant-negative effect, leading to structural defects and reduced stability in the microfibrils (Deng et al., 2025). These compromised microfibrils are unable to effectively guide and facilitate the orderly aggregation and covalent cross-linking of tropoelastin, ultimately resulting in the formation of elastic fibers that are morphologically abnormal, structurally disorganized, and mechanically compromised, manifesting as diminished tissue elasticity and tensile strength (Mead et al., 2024). The prognosis for most CCA patients is generally favorable. Unlike Marfan syndrome (MFS), cardiovascular involvement is rare in CCA (Singh and Wanjari, 2022). Notably, although cardiovascular involvement is uncommon in CCA, recent case reports suggest an association between *FBN2* mutations and intracranial aneurysms, highlighting the gene’s potential role in cerebrovascular disease (Ridha et al., 2019).

The proband carries heterozygous mutations in both *MYLK* and *FBN2*. These mutations may synergistically disrupt vascular homeostasis through distinct pathways: the *MYLK* mutation impairs smooth muscle contractile function, reducing the vessel wall’s ability to withstand hemodynamic shear stress, while the *FBN2* mutation compromises elastic fiber integrity, exacerbating structural weakness (Wang et al., 2010; Zodanu et al., 2024). This “dual-pathway synergistic disruption” model may explain the patient’s severe phenotype of early-onset, multi-site aneurysms (Dreher et al., 2025). In contrast, her parents carry only a single mutation each (father: *FBN2*; mother: *MYLK*) and exhibit no phenotype, likely due to incomplete penetrance.

Intracranial aneurysms (IA) and thoracic aortic aneurysms/dissections (TAA/D) are life-threatening conditions. Despite occurring in different locations, they share similarities and correlations in pathogenesis, epidemiology, and genomics (Changez et al., 2025). Regarding age of onset, the incidence of both increases with age, predominantly affecting individuals over 40 years old, likely due to vascular aging, reduced elasticity, and cumulative damage (Berger et al., 2025; Caffes et al., 2021). In terms of sex distribution, IA incidence is higher in women within specific age groups, while TAA/D overall shows a higher incidence in men, although certain types are also significant in women (Cai et al., 2022; Cote et al., 2022). This suggests sex factors may play a role in both diseases, implying common underlying risk factors. Furthermore, some genetic syndromes (e.g., Marfan syndrome) increase the risk for both TAA/D and potentially IA, indicating that patients with specific genetic backgrounds may face a dual risk (Vornetti et al., 2023).

Both diseases involve structural abnormalities of the vessel wall. IA formation is associated with localized weakness of the intracranial arterial wall, often due to deficiency or disruption of the medial muscle layer and elastic fibers. TAA/D primarily results from degenerative changes in the aortic media, including elastic fiber fragmentation and collagen reduction (Xu et al., 2019; Annambhotla et al., 2008). These similar structural alterations may stem from common pathophysiological mechanisms such as genetic predisposition, hemodynamic stress, and inflammation. Mutations in certain genes affecting vascular wall structure and function (e.g., elastin and collagen genes) may increase the risk for both conditions (Cocciolone et al., 2018; Arteaga-Solis et al., 2000).

Regarding genomic research, the pathogenesis of IA is not fully understood but is recognized as a complex disease involving genetic and environmental risk factors (Xu et al., 2019). Studies have implicated numerous genes in IA susceptibility and development, including *ACE*, elastin (*ELN*), endoglin (*ENG*), *COL3A1*, matrix metalloproteinase genes (*MMPs*), endothelial nitric oxide synthase (*eNOS/NOS*3), interleukin genes (*ILs*), tumor necrosis factor-alpha (*TNF-α*), apolipoprotein genes (*APOEs*), *PRDM*, *ANRIL*, and methylenetetrahydrofolate reductase (*MTHFR*) (Toader et al., 2023; Tromp et al., 2014). Significant progress has also been made in identifying genetic factors for TAA/D, with at least 31 candidate genes established for hereditary forms (e.g., *ACTA2*, *COL3A1*). The development of next-generation sequencing (NGS) continues to expand the genetic spectrum of affected patients (MILEWICZ et al., 2021).

This study has several limitations that should be acknowledged. First, the assessment of pathogenicity for the novel *FBN2* (NM_001999.4): c.1478A>G (p.Gln493Arg) and *MYLK* (NM_053025.4): c.834_835insGTA (p.Val278dup) variants relies primarily on *in silico* prediction tools (e.g., LRT, MutationTaster), which have inherent limitations in accurately predicting functional consequences *in vivo*; thus, our pathogenicity claims remain preliminary without further functional validation. Although literature suggests *FBN2* mutations may disrupt elastic fiber assembly and *MYLK* mutations could impair smooth muscle contraction, biochemical evidence is still needed for these specific variants (Mahdizadehi et al., 2023; Khapchaev and Shirinsky, 2016; Hannuksela et al., 2016). Second, the genetic analysis is constrained by the small family cohort (only the proband and parents); although we attempted to recruit additional relatives (grandparents and siblings) for segregation analysis, they were unavailable due to geographical and personal constraints. Third, while whole-exome sequencing (WES) was selected for its cost-effectiveness and efficiency in detecting coding variants in known vascular genes, it has limited sensitivity for non-coding variants (e.g., promoter/enhancer mutations) and structural variants compared to whole-genome sequencing (WGS). To partially mitigate this, copy number variants (CNVs) analysis was performed and ruled out large deletions/duplications in other vascular-related genes. Despite these limitations, our findings contribute to the growing evidence of shared mechanisms between intracranial and aortic vascular diseases and highlight the importance of comprehensive genetic evaluation in such cases.

In conclusion, intracranial aneurysms and thoracic aortic aneurysms/dissections exhibit correlations in epidemiology, pathophysiology, diagnosis, and management. Deeper investigation into these relationships will enhance our understanding of the pathogenic mechanisms underlying both diseases, improve diagnostic accuracy and therapeutic outcomes, and ultimately facilitate the delivery of more personalized medical care for patients.

## 5 Conclusion

This case highlights the interrelationship between intracranial aneurysms and thoracic aortic aneurysms across pathological mechanisms, clinical phenotypes, epidemiological patterns, and genomic landscapes. Genetic testing identified compound heterozygous mutations in *MYLK* and *FBN2*, shedding light on a novel genetic basis for complex vascular phenotypes. These findings expand the known mutational spectrum of thoracic aortic aneurysm/dissection (TAAD) and intracranial aneurysms, underscoring the value of integrative genetic analysis in unexplained cases. Further exploration of these shared pathways will deepen our understanding of disease pathogenesis, providing critical insights for early diagnosis, risk stratification, and targeted therapy. With ongoing research and technological advancements, it is anticipated that more precise preventive and therapeutic strategies for these life-threatening vascular disorders will emerge.

## Data Availability

The original contributions presented in the study are included in the article/supplementary material, further inquiries can be directed to the corresponding authors.

## References

[B1] Abdelrahman A. Elgassim M. Babiker A. M. Umer W. Ahmed A. (2023). Intracranial hemorrhage with concurrent aortic dissection. Radiol. Case Rep. 18 (1), 45–48. 10.1016/j.radcr.2022.09.058 36324836 PMC9619329

[B2] Annambhotla S. Bourgeois S. Wang X. Lin P. H. Yao Q. Chen C. (2008). Recent advances in molecular mechanisms of abdominal aortic aneurysm formation. World J. Surg. 32 (6), 976–986. 10.1007/s00268-007-9456-x 18259804 PMC2927355

[B3] Arteaga-Solis E. Gayraud B. Ramirez F. (2000). Elastic and collagenous networks in vascular diseases. Cell Struct. Funct. 25 (2), 69–72. 10.1247/csf.25.69 10885576 PMC3053004

[B4] Berger T. Dumfarth J. Kreibich M. Minatoya K. Ziganshin B. A. Czerny M. (2025). Thoracic aortic aneurysm. Nat. Rev. Dis. Prim. 11 (1), 34. 10.1038/s41572-025-00617-2 40341396

[B5] Burger J. Bogunovic N. de Wagenaar N. P. Liu H. van Vliet N. Ijpma A. (2021). Molecular phenotyping and functional assessment of smooth muscle-like cells with pathogenic variants in aneurysm genes *ACTA2, MYH11, SMAD3* and *FBN1* . Hum. Mol. Genet. 30 (23), 2286–2299. 10.1093/hmg/ddab190 34244757 PMC8600030

[B6] Caffes N. Wenger N. Cannarsa G. Oliver J. Onwukwe C. Gandhi D. (2021). Unruptured cerebral aneurysms in elderly patients: key challenges and management. Ann. Med. 53 (1), 1839–1849. 10.1080/07853890.2021.1990393 34664535 PMC8530485

[B7] Cai Y. Liu Z. Jia C. Zhao J. Chai S. Li Z. (2022). Comparison of sex differences in outcomes of patients with aneurysmal subarachnoid hemorrhage: a single-center retrospective Study. Front. Neurology 13, 853513. 10.3389/fneur.2022.853513 35572942 PMC9103686

[B8] Changez M. I. K. Nasir A. Sonsino A. Jeoffrey S. M. Kalyanasundaram A. Zafar M. A. (2025). Genetic overlap of thoracic aortic aneurysms and intracranial aneurysms. Genes. 16 (2), 154. 10.3390/genes16020154 40004483 PMC11855647

[B9] Cocciolone A. J. Hawes J. Z. Staiculescu M. C. Johnson E. O. Murshed M. Wagenseil J. E. (2018). Elastin, arterial mechanics, and cardiovascular disease. Am. J. Physiology-Heart Circulatory Physiology 315 (2), H189–H205. 10.1152/ajpheart.00087.2018 29631368 PMC6139627

[B10] Cote C. L. De Waard D. Kivell M. Fagan A. Horne G. Hassan A. (2022). Sex differences in trends in incidence of thoracic aortic Aneurysm repair and aortic dissection: 2005-2015. CJC Open 4 (12), 1081–1089. 10.1016/j.cjco.2022.08.012 36562011 PMC9764113

[B11] Deng M. Shen F. Zheng Y. Liu C. Luo Z. Wu X. (2025). Synostosis of joints caused by mutant FBN2 is linked to the abnormalities and misdifferentiation of articular surface cells. Genet. Med. 27 (10), 101537. 10.1016/j.gim.2025.101537 40709559

[B12] Dreher L. Abdul Nabi H. Vandolah H. Brennan S. Bcharah G. Bcharah H. (2025). The expanding genetic architecture of arteriopathies: from canonical TAAD genes to emerging connective tissue and signaling pathways. Med. Sci. 13 (3), 155. 10.3390/medsci13030155 40981152 PMC12452550

[B13] Duarte V. E. Yousefzai R. Singh M. N. (2023). Genetically triggered thoracic aortic disease: who should be tested? Methodist DeBakey Cardiovasc. J. 19 (2), 24–28. 10.14797/mdcvj.1218 36910552 PMC10000331

[B14] Halim D. Brosens E. Muller F. Wangler M. F. Beaudet A. L. Lupski J. R. (2017). Loss-of-Function variants in MYLK cause recessive megacystis microcolon intestinal hypoperistalsis syndrome. Am. J. Hum. Genet. 101 (1), 123–129. 10.1016/j.ajhg.2017.05.011 28602422 PMC5501771

[B15] Hamilton M. (2011). “Pathophysiology of aortic dissection and connective tissue disorders,” in Mechanisms of vascular disease: a reference book for vascular specialists. Editors Fitridge R. Thompson M. (Adelaide (AU): University of Adelaide Press). Available online at: http://www.ncbi.nlm.nih.gov/books/NBK534274/May 8, 2025). 30485028

[B16] Hannuksela M. Stattin E.-L. Johansson B. Carlberg B. (2015). Screening for familial thoracic aortic aneurysms with aortic imaging does not detect all potential carriers of the disease. AORTA 03 (01), 1–8. 10.12945/j.aorta.2015.14-052 26798750 PMC4714932

[B17] Hannuksela M. Stattin E.-L. Klar J. Ameur A. Johansson B. Sörensen K. (2016). A novel variant in MYLK causes thoracic aortic dissections: genotypic and phenotypic description. BMC Med. Genet. 17 (1), 61. 10.1186/s12881-016-0326-y 27586135 PMC5008005

[B18] Humphrey J. D. Schwartz M. A. (2021). Vascular mechanobiology: homeostasis, adaptation, and disease. Annu. Rev. Biomed. Eng. 23 (1), 1–27. 10.1146/annurev-bioeng-092419-060810 34255994 PMC8719655

[B19] Isselbacher E. M. Lino Cardenas C. L. Lindsay M. E. (2016). Hereditary influence in thoracic aortic aneurysm and dissection. Circulation 133 (24), 2516–2528. 10.1161/CIRCULATIONAHA.116.009762 27297344 PMC5031368

[B20] Keravnou A. Bashiardes E. Michailidou K. Soteriou M. Moushi A. Cariolou M. (2018). Novel variants in the ACTA2 and MYH11 genes in a Cypriot family with thoracic aortic aneurysms: a case report. BMC Med. Genet. 19 (1), 208–8. 10.1186/s12881-018-0728-0 30526509 PMC6286578

[B21] Khapchaev A. Y. Shirinsky V. P. (2016). Myosin light chain kinase MYLK1: anatomy, interactions, functions, and regulation. Biochem. Mosc. 81 (13), 1676–1697. 10.1134/S000629791613006X 28260490

[B22] Liu Y. Koyutürk M. Barnholtz-Sloan J. S. Chance M. R. (2012). Gene interaction enrichment and network analysis to identify dysregulated pathways and their interactions in complex diseases. BMC Syst. Biol. 6 (1), 65. 10.1186/1752-0509-6-65 22694839 PMC3426489

[B23] Luyckx I. Proost D. Hendriks J. M. H. Saenen J. Van Craenenbroeck E. M. Vermeulen T. (2017). Two novel *MYLK* nonsense mutations causing thoracic aortic aneurysms/dissections in patients without apparent family history. Clin. Genet. 92 (4), 444–446. 10.1111/cge.13000 28401540

[B24] Mahdizadehi M. Saghaeian Jazi M. Mir S. M. Jafari S. M. (2023). Role of fibrilins in human cancer: a narrative review. Health Sci. Rep. 6 (7), e1434. 10.1002/hsr2.1434 37469709 PMC10353528

[B25] Mead T. J. Martin D. R. Wang L. W. Cain S. A. Gulec C. Cahill E. (2024). Proteolysis of fibrillin-2 microfibrils is essential for normal skeletal development. eLife 11, e71142. 10.7554/eLife.71142 35503090 PMC9064305

[B26] Milewicz D. M. Prakash S. K. Ramirez F. (2017). Therapeutics targeting drivers of thoracic aortic aneurysms and Acute aortic dissections: insights from predisposing genes and mouse models. Annu. Rev. Med. 68, 51–67. 10.1146/annurev-med-100415-022956 28099082 PMC5499376

[B27] Milewicz D. M. Guo D. Hostetler E. Marin I. Pinard A. C. Cecchi A. C. (2021). Update on the genetic risk for thoracic aortic aneurysms and acute aortic dissections: implications for clinical care. J. Cardiovasc. Surg. 62 (3), 203–210. 10.23736/S0021-9509.21.11816-6 33736427 PMC8513124

[B28] Ostberg N. Zafar M. Ziganshin B. Elefteriades J. A. (2020). The genetics of thoracic aortic aneurysms and dissection: a clinical perspective. Biomolecules 10 (2), 182. 10.3390/biom10020182 31991693 PMC7072177

[B29] Ridha M. Mintz J. Voetsch B. (2019). Recurrent cervicocephalic dissections are associated with variants in connective tissue disease genes (P2.3-060). Neurology 92 (15_Suppl. ment), P2.3-060–060. 10.1212/WNL.92.15_supplement.P2.3-060

[B30] Rombouts K. B. van Merrienboer T. A. R. Ket J. C. F. Bogunovic N. van der Velden J. Yeung K. K. (2022). The role of vascular smooth muscle cells in the development of aortic aneurysms and dissections. Eur. J. Clin. Investigation 52 (4), e13697. 10.1111/eci.13697 34698377 PMC9285394

[B31] Shalata A. Mahroom M. Milewicz D. M. Limin G. Kassum F. Badarna K. (2018). Fatal thoracic aortic aneurysm and dissection in a large family with a novel MYLK gene mutation: delineation of the clinical phenotype. Orphanet J. Rare Dis. 13 (1), 41. 10.1186/s13023-018-0769-7 29544503 PMC5856213

[B32] Singh J. Wanjari A. (2022). Cardiac complications in Marfan Syndrome: a review. Cureus 14 (9), e29800. 10.7759/cureus.29800 36340521 PMC9622027

[B33] Toader C. Eva L. Bratu B.-G. Covache-Busuioc R. A. Costin H. P. Dumitrascu D. I. (2023). Intracranial aneurysms and genetics: an extensive overview of genomic variations, underlying molecular dynamics, inflammatory indicators, and forward-looking insights. Brain Sci. 13 (10), 1454. 10.3390/brainsci13101454 37891822 PMC10605587

[B34] Tromp G. Weinsheimer S. Ronkainen A. Kuivaniemi H. (2014). Molecular basis and genetic predisposition to intracranial aneurysm. Ann. Med. 46 (8), 597–606. 10.3109/07853890.2014.949299 25117779 PMC4438354

[B35] Vornetti G. De Martino S. R. M. Baroni M. C. Rossi C. Seri M. Mariucci E. (2023). Prevalence of unruptured intracranial aneurysms in patients with Marfan syndrome: a cross-sectional study and meta-analysis. Eur. Stroke J. 8 (2), 501–507. 10.1177/23969873221149848 37231696 PMC10334184

[B36] Wallace S. E. Regalado E. S. Gong L. Janda A. L. Guo D. C. Russo C. F. (2019). MYLK pathogenic variants aortic disease presentation, pregnancy risk, and characterization of pathogenic missense variants. Genet. Med. 21 (1), 144–151. 10.1038/s41436-018-0038-0 29925964 PMC6400320

[B37] Wang L. Guo D. Cao J. Gong L. Kamm K. E. Regalado E. (2010). Mutations in Myosin Light chain kinase cause familial aortic dissections. Am. J. Hum. Genet. 87 (5), 701–707. 10.1016/j.ajhg.2010.10.006 21055718 PMC2978973

[B38] Xu Z. Rui Y.-N. Hagan J. P. Kim D. H. (2019). Intracranial aneurysms: pathology, genetics, and molecular mechanisms. NeuroMolecular Med. 21 (4), 325–343. 10.1007/s12017-019-08537-7 31055715 PMC6829066

[B39] Xu P. Li R. Huang S. Sun M. Liu J. Niu Y. (2020). A novel splicing mutation in the FBN2 gene in a family with congenital contractural arachnodactyly. Front. Genet. 11, 143. 10.3389/fgene.2020.00143 32184806 PMC7058790

[B40] Yamaguchi M. Akabane S. Niitsu H. Nakahara H. Toshida A. Mochizuki T. (2025). The usefulness of comprehensive genome profiling test in screening of Lynch syndrome independent of the conventional clinical screening or microsatellite instability tests. J. Hum. Genet. 70, 385–393. 10.1038/s10038-025-01345-x 40335734 PMC12289520

[B41] Ziganshin B. A. Elefteriades J. A. (2014). Surgical management of thoracoabdominal aneurysms. Heart 100 (20), 1577–1582. 10.1136/heartjnl-2013-305131 25092876

[B42] Zodanu G. Hwang J. Mehta Z. Sisniega C. Barsegian A. Kang X. (2024). High-Throughput genomics identify novel FBN1/2 variants in severe neonatal Marfan Syndrome and congenital heart defects. Int. J. Mol. Sci. 25 (10), 5469. 10.3390/ijms25105469 38791509 PMC11122089

